# Thermomechanical Performance Analysis of Novel Cement-Based Building Envelopes with Enhanced Passive Insulation Properties

**DOI:** 10.3390/ma15144925

**Published:** 2022-07-15

**Authors:** Jorge Marin-Montin, Eduardo Roque, Yading Xu, Branko Šavija, Juan Carlos Serrano-Ruiz, Francisco Montero-Chacón

**Affiliations:** 1 Materials and Sustainability Group, Department of Engineering, Universidad Loyola Andalucía, Avenida de las Universidades, s/n, 41704 Sevilla, Spain; jjmarin@uloyola.es (J.M.-M.); eroque@uloyola.es (E.R.); jcserrano@uloyola.es (J.C.S.-R.); 2 Microlab, Faculty of Civil Engineering and Geosciences, Delft University of Technology, 2628 CN Delft, The Netherlands; y.xu-5@tudelft.nl (Y.X.); b.savija@tudelft.nl (B.Š.)

**Keywords:** building envelopes, thermal insulation, energy management, cement, PCM, finite elements

## Abstract

The design of new insulating envelopes is a direct route towards energy efficient buildings. The combinations of novel materials, such as phase-change (PCM), and advanced manufacturing techniques, such as additive manufacturing, may harness important changes in the designing of building envelopes. In this work we propose a novel methodology for the design of cement-based building envelopes. Namely, we combined the use of a multiscale, multiphysical simulation framework with advanced synthesis techniques, such as the use of phase-change materials and additive manufacturing for the design of concrete envelopes with enhanced insulation properties. At the material scale, microencapsulated PCMs are added to a cementitious matrix to increase heat storage. Next, at the component level, we create novel designs for the blocks, here defined as HEXCEM, by means of additive manufacturing. The material and component design process is strongly supported on heat transfer simulations with the use of the finite element method. Effective thermal properties of the mixes can be obtained and subsequently used in macroscale simulations to account for the effect of the volume fraction of PCMs. From the experimental and numerical tests, we report an increase in the the thermal inertia, which results in thermal comfort indoors.

## 1. Introduction

The building sector is responsible for 40% of the primary energy requirements in the EU and 36% of greenhouse gas emissions in Europe [1,2]. Therefore, there is a global need for improving energy management in buildings, which can be approached using different strategies [3] dealing with generation, energy storage, materials, or control systems that not only are more sustainable and environmentally-friendly, but also economical. One solution for providing thermal comfort while reducing the energy demand is the use of new insulating building envelopes [4,5].

Active/passive thermal energy management systems [6,7] based on renewable sources (e.g., solar radiation) are gaining much attention within the nearly zero energy buildings (NZEBs) paradigm [8]. While active systems are generally more difficult to implement in a building, passive systems can be easily integrated in building envelopes [9], with neither aesthetic nor functional drawbacks [7].

Concrete is one the most widely used materials worldwide [10,11]. It is mainly used in buildings for structural elements (i.e., columns and beams), foundations, slabs, and envelopes. Although concrete has caught attention for its mechanical properties, it can also be designed with targeted thermal insulation properties. For instance, higher internal porosity yields lower effective thermal conductivity [12]. Of course, this is a microstructural feature, but the same relationship applies when dealing with components. In this sense, if we place air gaps within a concrete block, the effective thermal conductivity of the component drops [13].

Another way to increase concrete’s thermal insulation properties is by means of so-called phase-change materials (PCMs). PCMs can absorb energy in the form of latent heat to complete a reversible solid-to-liquid phase transition [14,15]. In eutectic PCMs, this process takes place at a constant temperature. PCMs can be mixed with cementitious materials [16,17,18,19,20], and the resulting composite material has an increased heat capacity and better insulation properties, which are suitable for building envelopes [21,22]. In such cases, when the outdoor temperature increases, the material absorbs the heat, and if the melting point is surpassed, part of this energy is stored in the form of latent heat. This slows down the global heating process and the other way around.

PCMs themselves have low thermal conductivity [23]. Therefore, when embedded in the cement paste, the resulting thermal conductivity of the mix decreases [24,25]. This combination of low conductivity and high thermal energy storage capacity is very interesting in temperature management of buildings [26,27].

The integration of PCMs in cement-based materials is generally accomplished by encapsulation [28]. In the case of macrocapsules, they are generally placed in the mold before the casting [26] or mixed with the paste [14]. With the former method, a larger mass of PCM can be added and the risk of leakage is lower than that of the latter. One important drawback of micro-encapsulated PCMs is that it lowers the mechanical strength of the mortar in a drastic way [24]. However, the surface-area–volume ratio is much higher in microcapsules, which enhances the heat transfer process. Macrocapsules also may present partial phase transitions due to the low conductivity of the PCM and the larger sizes, which results in low performance of the energy storage feature [14].

Most of the works in the literature tackle the insulation design of building envelopes by means of PCMs [29]. These include experimental [30,31,32,33] and numerical approaches [34,35,36,37], mainly based on the use finite element method (FEM). Moreover, the FEM becomes a powerful method when dealing with coupled physical phenomena, such as heat transfer and solid mechanics [38,39]. However, there the coupled effect between PCMs and air voids have not been sufficiently covered. Wu et al. [40] presented a study on cellular cementitious composites with microencapsulated PCMs from a mechanical perspective. In this work, we focus on its thermomechanical behavior instead.

The design of building envelopes with PCMs and air cells is a multiscale problem. There exist different multiscale approaches [41] for solving thermomechanical problems. Homogenization-based schemes either by averaged properties [42] or so-called FE^2^ techniques [43,44,45] have been successfully used in these applications.

In this work we present a novel methodology for the design of cement-based building envelopes that combines the use of a multiscale, multiphysical simulation framework with advanced synthesis techniques, such as the use of phase-change materials and additive manufacturing. To improve the insulation properties, we tackled the design process at the material level, by means of microencapsulated PCMs, and the component level, via air cells. Moreover, additive printing (also commonly referred to as 3D printing) was used to prepare tailor-made specimens that were already analyzed numerically. Thus, we address the design process of building envelopes by combining advanced numerical and experimental techniques in a seamlessly fashion. In the first place, we designed the mix of the material and validated it numerically. Then, we used a homogenization approach to obtain macroscopic material properties that are used in FEM simulations of different envelope configurations. The numerical results are in perfect agreement with the experiments, fulfilling the validation process of the design and also validating a numerical framework to explore other designs that were not accounted for within the experimental campaign.

Although the design methodology presented in this paper focuses on building envelopes, it can be used without loss of generality in the design processes of other type of devices, such as thermal batteries [46], geothermal dissipation devices [47], freeze–thaw damage-resistant devices [48], or early-age cracking mitigation devices [16].

## 2. Materials and Methods

In this section we present the multiscale methodology followed for the design and validation of the building envelopes. In the first place, we designed a cementitious matrix with microencapsulated PCM at different volume fractions. These mixes were then characterized numerically using the FEM. The resulting material properties were subsequently upscaled. Then, we carried out the component design by considering different cell sizes. We used the FEM at the component scale for two purposes: (i) to validate the designs and (ii) to develop a multiscale simulation framework for new building envelopes.

### 2.1. Material Design

**Figure 1:**
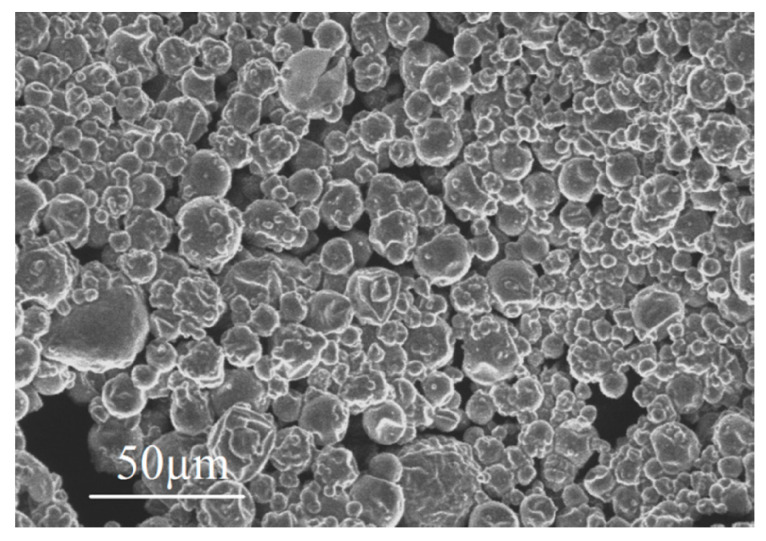
Micrograph of dispersed PCM microcapsules (reprinted from [49]). PCMs from the same batch were used in the current work.

The material designed for the building envelopes consists of a CEM I 42.5 cementitious matrix (ENCI, The Netherlands) and microencapsulated PCM (Encapsys LLC, United States) with different volume fractions: $V_{f}$ = 0%, 10%, and 30%. The mix had a water-to-cement ratio of w/c = 0.45 and PCM capsules reach a random distribution within the cement paste, as shown in Figure 1. The PCMs used are organic paraffin encapsulated by a melamine-formaldehyde shell. The same PCMs have been used by the authors in reference [49]. Further details on the PCM characterization can be found in references [49,50]. Table 1 shows the composition of the three mixes.

Once ready, the mixes were poured into the molds of the bricks and hardened for 2 days. Then, these were demolded and placed in a cure room until 21 or 28 days. According to [18], the thermal conductivity of cement paste decreases during the first 7 days of curing, but remains practically constant afterwards.

The PCMs used are organic paraffin encapsulated by a melamine-formaldehyde shell, as in reference [16]. The heat of fusion, $h_{f}$, is 143.5 J/g and the melting point, $T_{f}$, is 19 °C [49]. The thermophysical properties of the constituents are shown in Table 2.

#### 2.1.1. Unit Cell FEM Model

**Figure 2:**
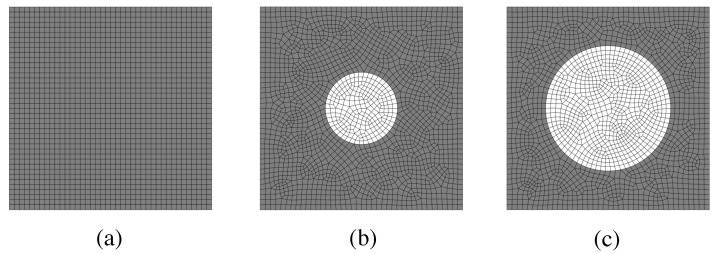
Mesh for different unit cells: (**a**) $V_{f}$ = 0%, (**b**) $V_{f}$ = 10%, and (**c**) $V_{f}$ = 30%.

In our modeling approach, we considered 2D unit cells of the material including the PCM and the cementitious matrix, which were explicitly modeled at different volume fractions. For the FEM discretization, we considered isoparametric quadrilateral elements. Figure 2 shows three different meshes. The material properties considered for each phase were those from Table 2.

Heat transfer is described as an elliptical partial differential equation that reads:

(1) $$\rho c_{p}\frac{\partial T}{\partial t}-\nabla \cdot \left(k\nabla T\right)=q$$

where ρ is the density, $c_{p}$ the specific heat, *T* is the temperature field, *t* is time, *k* is the thermal conductivity, and *q* is the volumetric heat source.

In this work, we account for the non-linear effect of the phase transition following the effective specific heat approach [51,52]:

(2) $$c_{p}\left(T\right)=\left\{\begin{array}{cc} c_{p,s} & \mathrm{for}\;T<T_{f}-\frac{\Delta T_{f}}{2}\\ c_{p,s}+\frac{h_{f}}{\Delta T_{f}} & \mathrm{for}\;T_{f}-\frac{\Delta T_{f}}{2}\leq T\leq T_{f}+\frac{\Delta T_{f}}{2}\\ c_{p,l} & \mathrm{for}\;T>T_{f}-\frac{\Delta T_{f}}{2} \end{array}\right.$$

where $c_{p,s}$ and $c_{p,l}$ are the specific heat in the solid and liquid phases, respectively, $h_{f}$ is the enthalphy of fusion, $T_{f}$ is the phase-change temperature, and $\Delta T_{f}$ the temperature window.

**Figure 3:**
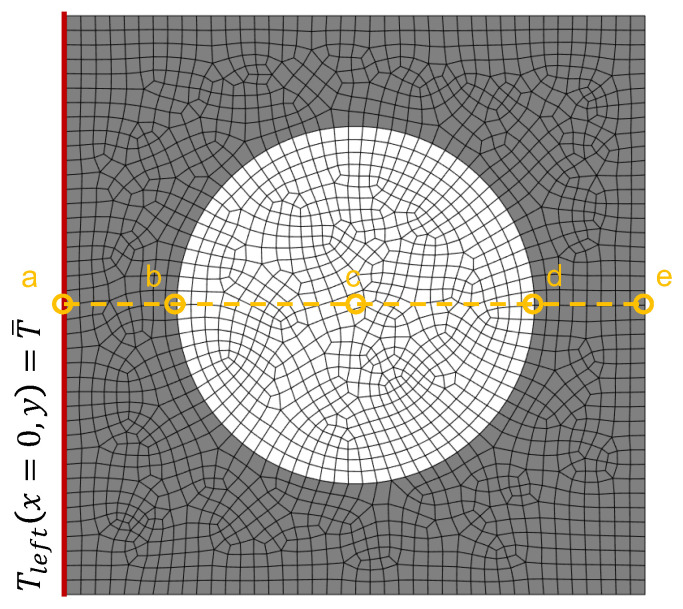
Boundary conditions of the unit cell problem. The centerline indicates different probe locations.

In order to solve an initial boundary value problem (IBVP), apart from the partial differential equations involved, we need to define the solution domain (i.e., the unit cell and time) and initial and boundary conditions. In our simulations we applied Dirichlet boundary conditions on the left side of the unit cell and evaluated the temperature evolution at different locations (Figure 3).

The heat transfer problem can be discretized using the FEM [53], yielding the following matrix form:

(3) $$C_{th}\dot{T}+K_{th}T=q$$

where $C_{th}$ and $K_{th}$ are the heat capacity and thermal conductivity matrices, respectively, T is the temperature vector, and q is the flux vector.

This is a time-dependent problem; therefore, we can discretize in time using Equation (3) with a time increment Δt, such that at an instant of time $t_{j}$, the system reads:

(4) $$C_{th}{\dot{T}}_{j}+K_{th}T_{j}=q_{j}$$

We make use of the Crank–Nicolson time integration scheme [53] to determine the temperature at an instant of time $t_{j+1}$:

(5) $$T_{j+1}={\left(\frac{1}{\Delta t}C_{th}+\frac{1}{2}K_{th}\right)}^{-1}\left[\left(\frac{1}{\Delta t}C_{th}-\frac{1}{2}K_{th}\right)T_{j}+q\right]$$

Since this problem is non-linear, we must update the capacity matrix at each time step, i.e., $C_{th}=C_{th}\left(T_{j}\right)$, which gathers the phase-change phenomenon, as described above.

#### 2.1.2. Upscaling Procedure

The unit cell model is heterogeneous, since it accounts for two different phases. However, for the component design, we require material properties of the mix. For this purpose, we use an upscaling homogenization approach [54] for determining the macroscopic thermophysical properties, namely, *k* and $\rho c_{p}$.

The effective conductivity, $k^{eff}$, of a two-phase system with spherical inclusions can be obtained as [55]:

(6) $$k^{eff}=\frac{2k^{m}\left(1-\phi \right)+\left(1+2\phi \right)k^{p}}{\left(2+\phi \right)+\left(1-\phi \right)\frac{k^{p}}{k^{m}}}$$

where $k^{m}$ and $k^{p}$ are the thermal conductivity of the matrix and particle, respectively, and ϕ is the volume fraction of particles. In our case, $V_{f}=\phi$, so we keep using ϕ for the mathematical description of the problem in the following.

The effective volumetric heat capacity, $\rho c_{p}^{eff}$, is evaluated using mixing rule [52]:

(7) $$\rho c_{p}^{eff}\left(T\right)=\phi \left(\rho^{p}c_{p}^{p}\left(T\right)\right)+\left(1-\phi \right)\left(\rho^{m}c_{p}^{m}\left(T\right)\right)$$

where $\rho^{m}$ and $\rho^{p}$ are the densities of the matrix and particle, respectively. Similarly, $c_{p}^{m}$ and $c_{p}^{p}$ are the specific heats of the matrix and particle, respectively.

Using the definitions above, it is possible to set the homogenized phase-change (i.e., temperature-dependent) model as:

(8) $$\rho c_{p}^{eff}\left(T\right)=\left\{\begin{array}{cc} \rho c_{p,s}^{eff} & \mathrm{for}\;T<T_{f}-\frac{\Delta T_{f}}{2}\\ \rho c_{p,s}^{eff}+\phi \rho^{p}\frac{h_{f}}{\Delta T_{f}} & \mathrm{for}\;T_{f}-\frac{\Delta T_{f}}{2}\leq T\leq T_{f}+\frac{\Delta T_{f}}{2}\\ \rho c_{p,l}^{eff} & \mathrm{for}\;T>T_{f}-\frac{\Delta T_{f}}{2} \end{array}\right.$$

where $\rho c_{p,s}^{eff}$ and $\rho c_{p,l}^{eff}$ are the effective volumetric heat capacity of the solid and liquid phases, respectively, $\rho^{p}$ is the density of the particle, and $h_{f}$ and $T_{f}$ are the heat of fusion and melting point of the PCM.

### 2.2. Component Design

The new building envelope proposed herein is made of a PCM-based cementitious matrix, described previously, with air cells, yielding a honeycomb-like structure. In the first place, tailor-made molds were fabricated using 3D printing technology. Then, different brick configurations were cast and tested experimentally using a hot plate setup. Finally, the designs were validated using a macroscopic FEM model. Moreover, the simulation framework was also validated experimentally so that it can be used in the analysis of different designs.

#### 2.2.1. Experimental Methods

**Figure 4:**
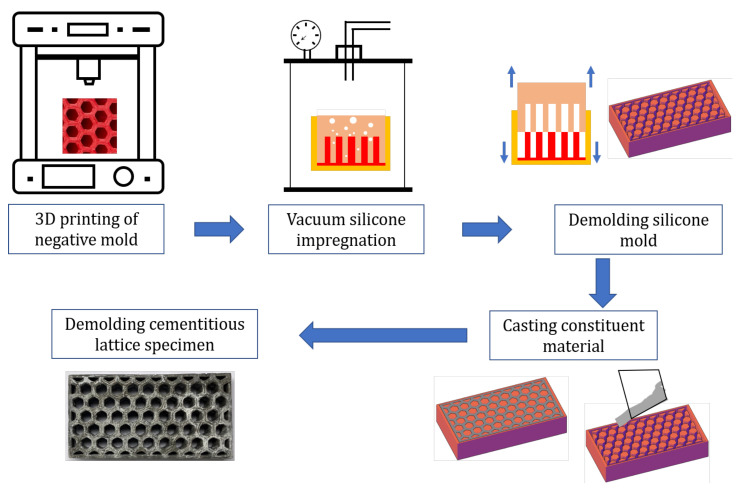
Summary of the specimens production based on CAD and additive manufacturing techniques.

The building envelopes follow a honeycomb structure so that large amounts of air could be hosted within the blocks while preserving a compact and robust structure. Due to the complex geometry of the blocks, new tailor-made molds were fabricated using 3D printing technology [56] (Ultimaker 2 + 3D) with acylonitrile butadiene styrene (ABS) and polylactic acid (PLA). Namely, we used ABS for the large air cells design, and we used PLA for the other two. The advantage of PLA over ABS is that the former is biodegradable and allows a higher print speed [57]. In any case, the choice of the material does not affect the results of this research. Three blocks with size 100×52×20 mm^3^ and different air cell sizes were designed using CAD techniques (Figure 4).

**Figure 5:**
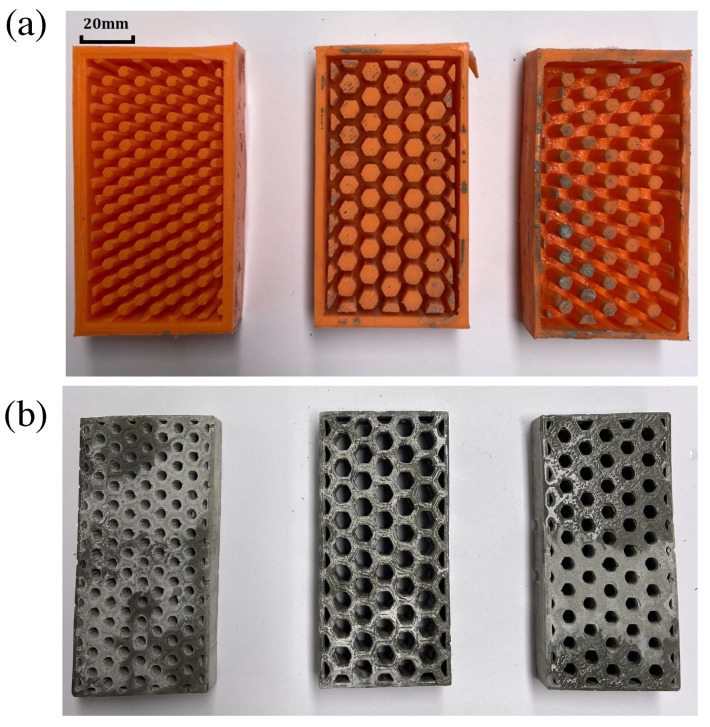
(**a**) Molds of the bricks. (**b**) Cast specimens.

Once the bricks were printed, these were filled with a mix of two components of PS8510 silicon rubber, which takes two hours to harden. In such a way, molds of the printed bricks were realized in a flexible material, which is better for the demolding process (Figure 5a). Another option is to directly print the molds in 3D, but this may result in lower precision [58].

Finally, three types of honeycomb blocks (i.e., small, medium, and large cells), as shown in Figure 5b, were cast using the material mix described previously. Ordinary cement paste (CEM I 42.5, w/c = 0.45) included encapsulated organic paraffin wax with different volume fractions ($V_{f}$ = 0%, 10%, and 30%). Table 3 shows the different configurations considered in our analysis.

**Figure 6:**
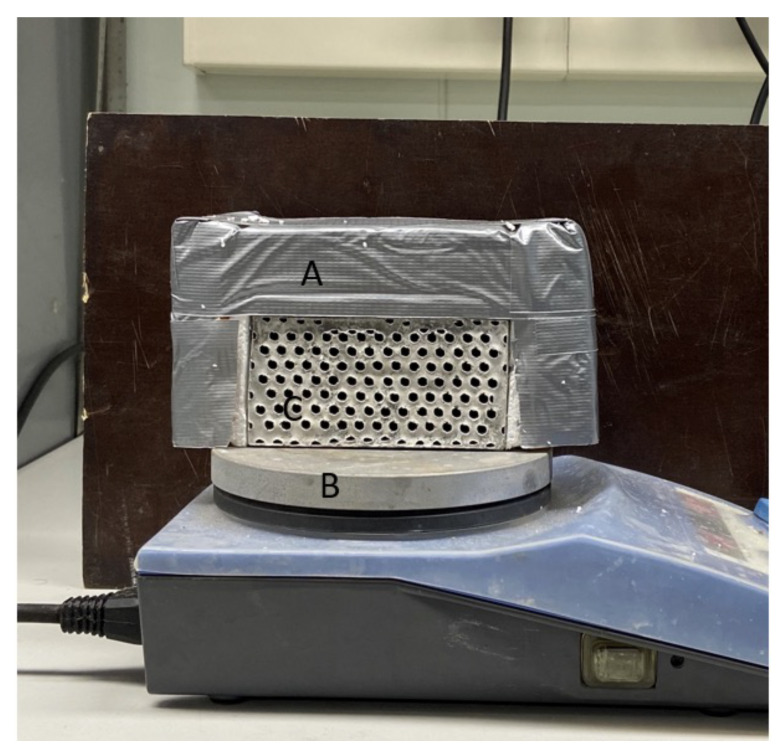
Hot plate setup. Labels: (A) insulation, (B) hot plate, (C) specimen.

To analyze the thermal behavior of the new blocks, a hot plate setup (Figure 6) was used along with a FLIR A320 thermal camera and ThermaCAM Researcher Pro software. All the specimens were insulated on the free boundaries (i.e., back, top, and sides) except for the front, which was left open so as to evaluate the temperature evolution with the thermal camera. The insulation was made of styrofoam, sponges, and duct tape. The hot plate was set to 100 °C, and the room temperature was 22.5 ± 1 °C. The tests had a total duration of 3.5 h.

The evolution of the temperature was obtained for three sample heights, namely, 30%, 60%, and 90%, to see the spatial gradient. Temperature was evaluated every 9 min until the steady-state conditions were accomplished.

#### 2.2.2. Brick FEM Model

At the component level, we used the homogenized phase-change FEM formulation presented in Section 2.1.2 for the non-linear unsteady heat transfer problem. For the FEM model, we used isoparametric quadrilateral elements, and the boundary conditions reflected those of the hot plate setup.

**Figure 7:**
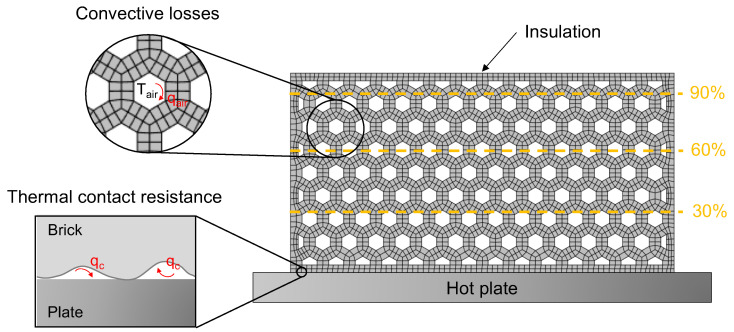
Boundary conditions of the component FEM model and probe locations.

As shown in Figure 7, the contact of the bottom side of the brick with the hot plate was not perfect, and internal convective losses due to the surface roughness appeared. In order to model this, we used thermal contact resistance elements (i.e., interfacial conductance) that were calibrated with part of the experimental results. The temperature of the source was set to that of the hot plate, 100 °C. Air trapped in the cells would also generate convective losses that would affect the heat transfer process in the blocks. These were taken into account in our model by using film conditions:

(9) $$\begin{array}{c} q_{bottom,j}=h_{c}A_{bottom}\left(T_{plate}-T_{bottom,j}\right)\\ q_{cells,j}=h_{air}A_{cells}\left(T_{air}-T_{cells,j}\right) \end{array}$$

where $q_{bottom,j}$ is the imposed flux at the bottom nodes and instant $t_{j}$, $h_{c}$ is the interfacial conductance, $A_{bottom}$ is the contact surface, $T_{plate}$ is the temperature of the hot plate, $T_{bottom,j}$ is the temperature of the bottom surface of the brick, $q_{cells,j}$ is the imposed flux on the inner surface of the cells, $A_{cells}$ is the inner surface of the cells, $T_{air}$ is the air temperature at the cells, and $T_{cells,j}$ is the temperature at the inner surface of the cells.

The time evolution of the temperature distribution was analyzed by placing three probes in the domain at the center of the horizontal axis, and three different heights: 30%, 60%, and 90%—those recorded in the experimental setup.

We extended our analysis of the component so as to evaluate the mechanical stresses promoted by the temperature gradients. Thus, according to the constitutive equation in solid mechanics:

(10) $$\sigma =D:\varepsilon^{el}$$

where σ is the stress tensor, D the consitutive tensor, and ε the elastic strain tensor.

The elastic strain tensor can be evaluated as:

(11) $$\varepsilon^{el}=\varepsilon -\varepsilon^{th}$$

where ε is the total strain obtained from the displacement field gradient, and $\varepsilon^{th}$ is the thermal strain tensor, which in isotropic solids is:

(12) $$\varepsilon^{th}=\alpha \Delta TI$$

where α is the thermal expansion coefficient; ΔT is the temperature jump defined as the difference between the temperature field obtained from the heat transfer analysis, *T*, and the reference temperature, $T_{r}$, i.e., $\Delta T=T-T_{r}$; and I is the identity tensor.

Regarding the FEM model, we used isoparametric quadrilateral elements for the space discretization, and the system was solved at every instant of time $t_{j}$:

(13) $$Ku_{j}=f_{j}$$

where K is the stiffness matrix, $u_{j}$ the displacement vector, and $f_{j}$ the force vector, which is defined as:

(14) $$f_{j}=f_{m,j}+f_{th,j}$$

with $f_{m,j}$ being the mechanical force vector and $f_{th,j}$ the thermal force vector.

For an element *el* and isotropic expansion, the element thermal force vector, $f_{th,j}^{el}$, reads:

(15) $$f_{th,j}^{el}=\alpha \Delta TB^{T}D{\left[\begin{array}{ccc} 1 & 1 & 0 \end{array}\right]}^{T}$$

B is the matrix containing first-derivatives of the shape functions, and D is the constitutive matrix (in our case, plane stress problem).

Finally, we solved the mechanical problem for each $t_{j}$ using the preconditioned conjugate gradient method.

## 3. Results and Discussion

### 3.1. Material Thermal Characterization

In the first place, we performed heat transfer transient simulations at the unit cell scale to characterize the thermal behavior of the two materials designs. For this purpose, we defined three unit cells with the following volume fractions of PCM: $V_{f}=0\%,10\%,$ and 30%. The size of the unit cell was 10 µm, and the capsule sizes were 3.5 and 6 µm for the 10 and 30% contents, respectively, which are in the ranges of similar compositions [59,60,61]. The FEM mesh consisted of approximately 2000 elements.

**Figure 8:**
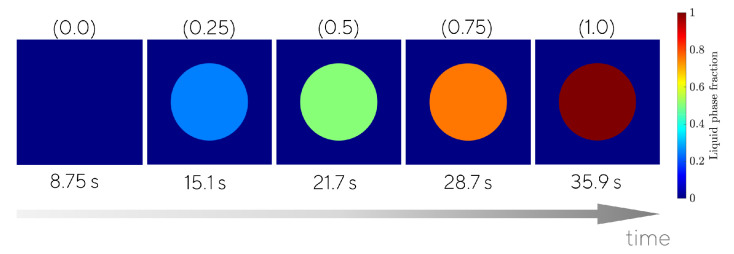
Phase transition at the unit cell problem ($V_{f}$ = 30%. Top label (in parentheses): Average liquid fraction. Bottom label: time.

The unit cells were initially set to T(x,0)=15 °C, and the temperature at the left edge, x=0, identified with the label “a” in Figure 3, was suddenly set to T(0,y,t)=30 °C. The non-linear heat transfer problem was solved for the first 200 s. As observed in Figure 8, at the microscale it took approximately 36 s to complete the phase transition, which in our paraffin wax takes place at $T_{f}=19$ °C. We have considered a temperature window of $\Delta T_{f}=2$ °C and the thermophysical properties in Table 2.

**Figure 9:**
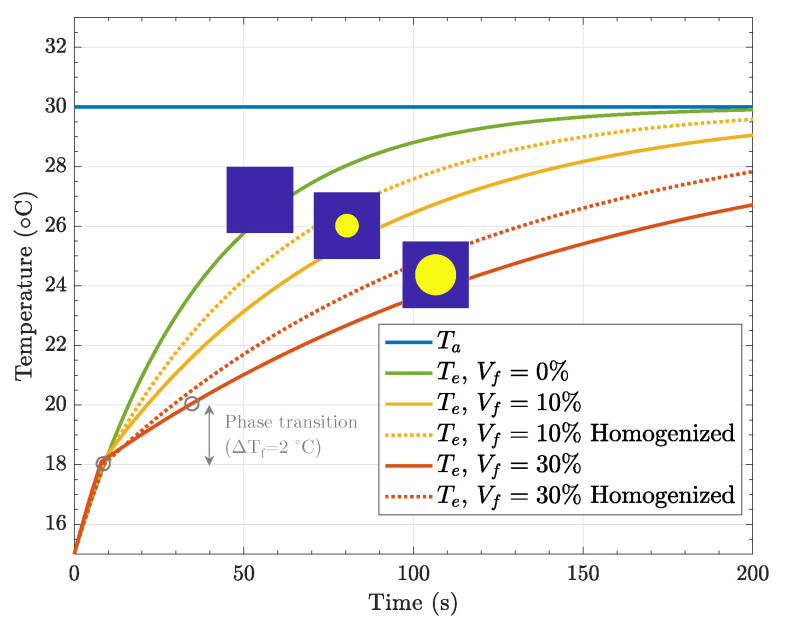
Temperature evolution of different compositions obtained for the heterogeneous model and compared to homogenized properties.

Figure 9 shows the evolution of the temperature of the points labeled as *a* and *e* as per Figure 3, i.e., the left and right edges of the unit cell. We used two types of model: (i) a heterogeneous model accounting explicitly for the phase-change phenomenon in the PCM particles (results are shown as continuous lines in Figure 9), and (ii) a homogeneous model using the homogenization approach presented in the previous section (results are shown as dotted lines in Figure 9). We can observe how the temperature rises during the first 9 s from 15 °C to 18 °C, where the phase transition starts taking place in our approach (we use a temperature window of ±1 °C with respect to the melting point ($T_{f}=19$ °C). Thus, we observed a change in the slope of the curves when the phase transition, solid to liquid, started.

In the case of no PCMs ($V_{f}=0\%$), it can be seen that there was no change in slope, and the temperature at the right edge reached that of the one imposed at the left edge at 200 s. This means that steady-state conditions were reached. As we increased $V_{f}$, we observed that the material required more time to achieve these conditions. Thus, the addition of PCM delays the rising of temperature, and we also confirmed that the higher the PCM amount, the higher the thermal inertia exhibited by the composite material. In fact, with the heterogeneous model, we measured 1.8× delay until reaching 95% of the steady-state conditions for $V_{f}=10\%$, and this number increased up to 3.4× in the case of $V_{f}=30\%$. We also observed some differences between the homogeneous model, in which one phase with homogenized thermophysical properties was modeled, and the heterogeneous one. These differences may have come from the fact that the homogenized expressions correspond to a 3D case (inclusions were treated as spheres), whereas the heterogeneous model considered inclusions as disks. In any case, the same meshes were used for both models. With the the homogenized model being more conservative in terms of thermal management capabilities, we used it in the brick simulation.

### 3.2. Brick Characterization

**Figure 10:**
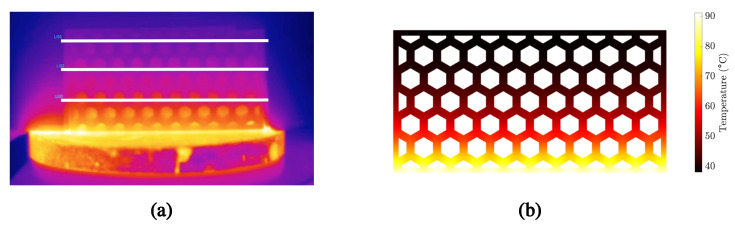
Temperature distribution within the brick during the hot plate test: (**a**) Thermographic analysis. (**b**) FEM simulations.

Once the new mix (i.e., PCM and cement) had been characterized, we proceeded with the characterization of the building envelopes. For this reason, we tested different designs (summarized in Table 3) using the hot plate setup. We also performed FEM simulations of the bricks to better understand the effects of $V_{f}$ and $\phi_{air}$ on the global insulating properties of the bricks. As pointed out in Section 2.2.1, thermographic analysis was used to measure the temperature evolution within the brick. Figure 10 shows the temperature distribution obtained through thermographic analysis and the corresponding estimates using the FEM model with homogenized properties.

**Figure 11:**
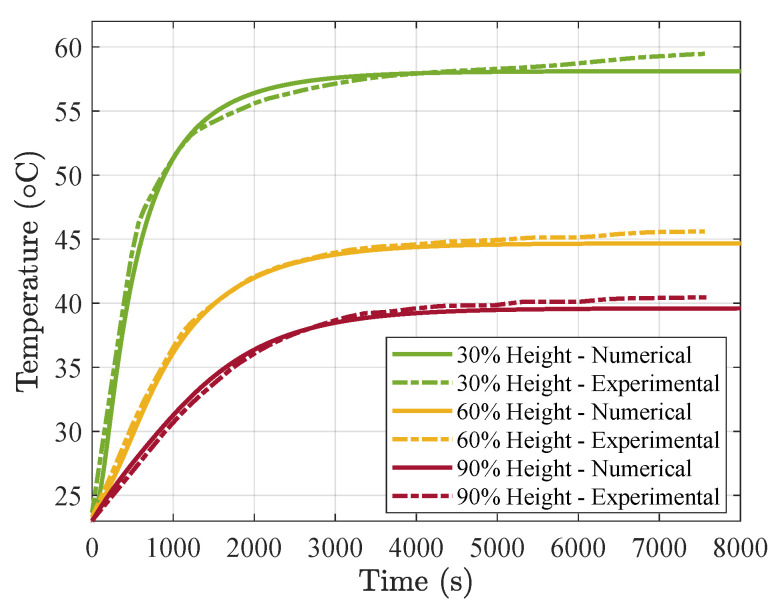
Numerical vs. experimental temperature evolution for $V_{f}=0\%$ and large air cells at different heights of the brick.

As a first step in our modeling tasks, we needed to calibrate the thermal conductance of the brick-plate interface. In Figure 11 we show the experimental results (in dotted lines) and the equivalent simulation results (in continuous lines) of the large air cell model, which was the one used for the calibration, since we have it for the three different grades of PCM. From the results, we can observe that a conductance of $h_{c}=200$ W/m^2^K provides a good fit. This value of *h_c_* is in agreement with that observed for concrete–steel interfaces [62]. We considered an air film coefficient of $h_{air}=10$ W/m^2^K.

#### 3.2.1. Effect of *V_f_*

**Figure 12:**
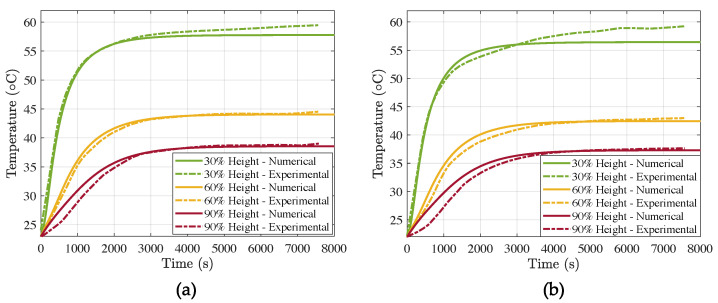
Numerical vs. experimental temperature evolution for large air cells at different heights of the brick: (**a**) $V_{f}=10\%$ and (**b**) $V_{f}=30\%$.

In Figure 12 we show the temperature evolution during ca. 2.5 h, for the large air cell specimens with $V_{f}=10\%$ (Figure 12a) and $V_{f}=30\%$ (Figure 12b). In the first place, we can observe that the increase in PCM content provided higher thermal inertia and insulating properties. In fact, during the first 3000 s, the temperature rise in the mix with higher $V_{f}$ increased at a lower pace than that of $V_{f}=10\%$. The steady-state conditions were reached after close to 4000 s.

If we look at the 90% height, which is the most interesting line, for it is closest to the indoor one, we can observe again that after 1000 s, the difference in temperature was about 2.5 °C between both mixes. The steady-state conditions were also milder in the case of $V_{f}=30\%$.

#### 3.2.2. Effect of Air Cells

**Figure 13:**
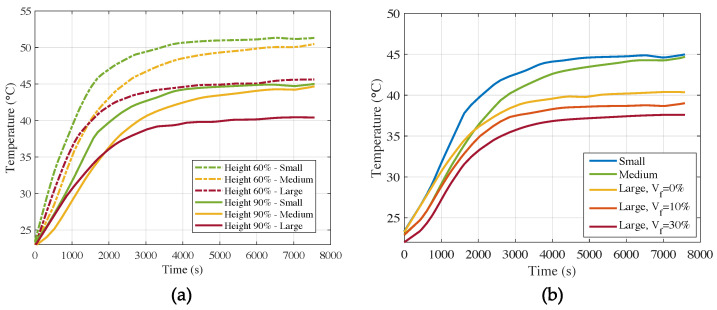
Experimental temperature evolution of: (**a**) $V_{f}=0\%$ and (**b**) All specimens at 90%.

We now study the effect of air cells in the insulating properties of the bricks. In Figure 13a it can be seen that at 60% of the height of the samples without PCM, the temperature difference after 6000 s was greatest between the samples with the small air cells and the large air cells. The sample with the large air cells heated up the slowest, as expected, since the volume of the air cells covered 44% of the total volume of the cementitious sample, whereas the small air cells and the medium air cells only covered 30% and 24% of the total volume, respectively. The fact that the sample with the medium-sized air cells heated up slower than the one with the small air cells does not seem to be in line with the literature. The same seems to happen in Figure 10, where the average temperature at 90% of the height of the samples is shown. The sample with small air cells heated up faster than the sample with medium air cells, which is not in proportion with the volume fraction of air within the different samples. Just like in the case of the PCM-containing samples, the graphs for 60% and 90% height are very similar in terms of shape and temperature difference between the samples.

In Figure 13b, it can be observed that larger hole specimens (L) presented a temperature decrease of at least 5 °C with respect to small hole specimens (S) when no PCM was added, and up to 7.5 °C with $V_{f}=30\%$.

#### 3.2.3. Thermomechanical Assessment

Finally, we used the temperature field obtained from the heat transfer simulations to run a sequential FEM of the mechanical problem. We considered an elastic modulus E=20 GPa, as observed by [49], a Poisson’s ratio ν=0.2, and thermal expansion coefficient $\alpha =10^{-5}$ 1/K. We ran the simulation for the first 3000 s, where the most important temperature gradients appeared.

**Figure 14:**
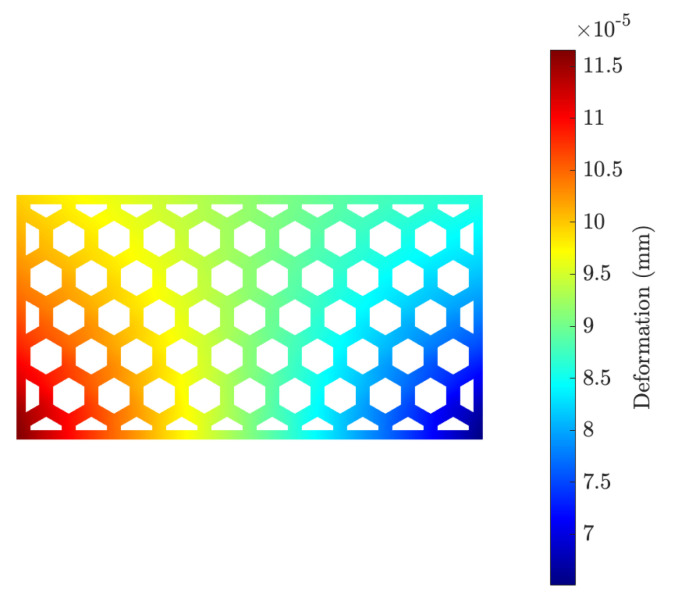
Total deformation in the brick after 3000 s.

In Figure 14, we show the total deformation of the brick, for large air cells and $V_{f}=10$%. One can see the free expansion deformation mode with the highest values at the bottom tips.

**Figure 15:**
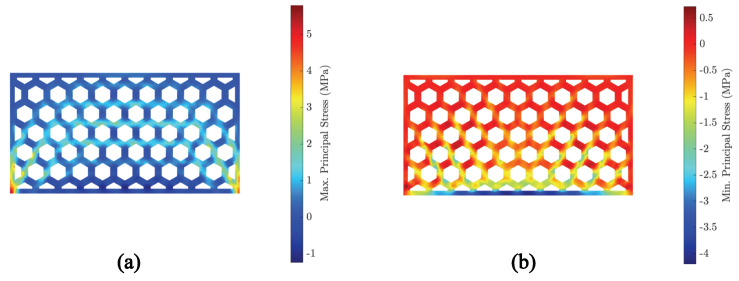
Principal stresses in the brick after 3000 s: (**a**) Maximum principal stress. (**b**) Minimum principal stress.

Figure 15 shows the maximum (Figure 15a) and minimum (Figure 15b) principal stresses at the same instant of time. As expected from the deformation field, the highest maximum principal stress appeared at tips of the bottom surface. It must be remarked that we considered sliding boundary conditions for the bottom surface. Apart from the stress concentration due to boundary conditions, we observed well-developed stress bands below the tensile strength (ca. 2 MPa). Regarding the minimum principal stresses, these are well below the compressive strength as measured in [49]. This is in agreement with the experimental observations, where no cracks were identified.

## 4. Conclusions

The design of new insulating envelopes is a direct route towards energy efficient buildings. The use of advanced numerical and manufacturing techniques may lead to important advances in this sense. In this work we combined in a seamless fashion new materials (e.g., PCMs), manufacturing (e.g., 3D printing), and simulation techniques (e.g., finite element method) in the design of new insulating components with enhanced properties. Namely, we presented the so-called HEXCEM component, which has important benefits, such as improved thermal insulation, reduced energy consumption and GHG emissions, reduced specific weight, and applicability (it could be also used in new buildings as well as in the rehabilitation of older buildings).

For this purpose, a new synthesis approach based on additive manufacturing of tailor-made molds and microencapsulated PCM has been successfully implemented. As we showed in this work, complex geometries can be easily accomplished.

In order to reinforce the design process for both the material mix and the component, a two-scale (material and component) simulation strategy has been successfully developed. For that, the numerical simulations carried out with our methodology showed excellent agreement with the experimental tests. Moreover, it can be used to explore new designs for different applications before actually synthesizing them.

From the analyses, we found that microencapsulated PCM in cement, within the phase-transition temperature, may lead to 1.8× delay to 95% steady-state conditions in the case of $V_{f}=10\%$, and 3.4× for $V_{f}=30\%$. On the other hand, larger hole specimens (L) present a temperature decrease of at least 5 °C with respect to small hole specimens (S) when no PCM is added, and up to 7.5 °C with $V_{f}$ = 30%.

With respect to the thermomechanical behavior, the simulations of the bricks showed no thermal cracking. This is also in agreement with the experimental observations. This is important for ensuring mechanical integrity.

## Figures and Tables

**Table 1:** Composition of the mixes (g/L) for different PCM volume fractions.

Constituent	$V_{f}$ = 0%	$V_{f}$ = 10%	$V_{f}$ = 30%
CEM I 42.5	1354.8	1219.8	948.6
water	610.2	548.8	426.9
PCM	0	90	270

**Table 2:** Thermophysical properties of the phases [16,49].

Material Property	Cement	Paraffin Wax
Density, ρ (kg/m^3^)	1965	900
Conductivity, *k* (W/mK)	1.00	0.42
Specific heat, *c_p_* (J/kgK)	1530	1900
Enthalpy of fusion, *h_f_* (J/kg)	n/a	143,500
Melting point, $T_{f}$ (°C)	n/a	19

**Table 3:** Building envelope configurations tested.

Reference	Size (mm)	$\phi_{air}$	ϕ	Curing Time (Days)
Large (L)	100×52×20	0.44	0.0, 0.1, 0.3	21, 21, 28
Medium (M)	100×52×20	0.24	0.0	21
Small (S)	100×52×20	0.30	0.0	21

## Data Availability

Not applicable.
